# The importance of baseline viral load when assessing relative efficacy in treatment-naïve HBeAg-positive chronic hepatitis B: a systematic review and network meta-analysis

**DOI:** 10.1186/2046-4053-3-21

**Published:** 2014-03-07

**Authors:** Stuart Mealing, Isabella Ghement, Neil Hawkins, David A Scott, Benedicte Lescrauwaet, Maureen Watt, Mark Thursz, Pietro Lampertico, Lorenzo Mantovani, Edith Morais, Bruno Bregman, Michel Cucherat

**Affiliations:** 1 Oxford Outcomes Ltd, Seacourt Tower, West Way, Oxford OX2 0JJ, UK; 2 Ghement Statistical Consulting Company Ltd, Richmond, Canada; 3 Xintera Consulting, Leuven, Belgium; 4 Department of medicine, Imperial College, London, UK; 5 1st Division of Gastroenterology, A.M. e A. Migliavacca Centre for the Study of Liver Disease, Milan, Italy; 6 School of Pharmacy at the University of Milan, Milan, Italy; 7 Bristol-Myers Squibb, Rueil-Malmaison, France; 8 University Claude Bernard Lyon 1, Lyon, France

**Keywords:** Network meta-analysis, Relative efficacy, Systematic review, Virologic response, Entecavir

## Abstract

**Background:**

To date no network meta-analysis (NMA) has accounted for baseline variations in viral load when assessing the relative efficacy of interventions for chronic hepatitis B (CHB). We undertook baseline-adjusted and unadjusted analyses using the same data to explore the impact of baseline viral load (BVL) on CHB treatment response.

**Methods:**

We searched Embase, Medline, Medline in Process and the Cochrane CENTRAL databases for randomised clinical trials (RCTs) of monotherapy interventions at licensed doses for use in CHB. Search strategies comprised CHB disease and drug terms (a combination of controlled vocabulary and free text terms) and also a bespoke RCT filter.

The NMA was undertaken in WinBUGs using fixed and random effects methods, using data obtained from a systematic review. Individual patient data (IPD) from an entecavir clinical trial were used to quantify the impact of different baseline characteristics (in particular undetectable viral load (UVL) at 1 year) on relative treatment effect. Study level mean baseline values from all identified studies were used. Results were generated for UVL and presented as relative risks (RRs) and 95% credible intervals (CrIs) using entecavir as reference treatment.

**Results:**

Overall, for all eight relevant interventions we identified 3,000 abstracts. Following full text review a total of 35 (including the contents of six clinical study reports) met the inclusion critera; 19 were in hepatitis B e antigen (HBeAg)-positive patients and 14 of the 19 contained outcome information of relevance to the NMA.

Entecavir and tenofovir studies had heterogeneous patient populations in terms of BVL (mean values 9.29 and 8.65 log_10_ copies/ml respectively). After adjusting UVL for BVL using an informative prior based on the IPD analysis, the difference between entecavir and tenofovir was not statistically significant (RR 1.27, 95% CrI 0.96 to 1.47 - fixed effects). A similar conclusion was found in all sensitivity analyses. Adjusted tenofovir results were more consistent with observed clinical trial response rates.

**Conclusions:**

This study demonstrates the importance of adjusting for BVL when assessing the relative efficacy of CHB interventions in achieving UVL. This has implications for both clinical and economic decision making.

## Background

Chronic hepatitis B (CHB) is responsible for about 600,000 deaths worldwide per year, from end-stage liver disease or hepatocellular carcinoma (HCC) [1]. An estimated 350 to 400 million people have CHB [2], of whom 15 to 40% will eventually experience serious complications (hepatic cirrhosis, hepatic decompensation or HCC) [3]. Development of complications is associated with persistent replication of the hepatitis B virus (HBV) [2]; hence, an important goal of CHB treatment is long-term suppression of HBV replication to undetectable levels, as measured by serum HBV DNA (virologic response) [2,4]. Normalization of serum alanine transaminase (ALT), loss of hepatitis B e antigen (HBeAg) and improvement in liver histology are other recognized measures of CHB treatment efficacy.

Current European clinical guidelines recommend the following treatment options for CHB: entecavir, lamivudine, telbivudine, adefovir dipivoxil, tenofovir dipivoxil fumarate, peginterferon-alfa-2a, interferon-alfa-2a, and interferon-alfa-2b [2]. Information on their relative efficacy is important in order for healthcare professionals and payers to make evidence-based decisions on which treatments to prescribe. Because head-to-head comparisons of competing CHB treatment options via randomised clinical trials (RCTs) are not available for all comparators in HBeAg antigen-positive or -negative CHB, indirect evidence in the form of network meta-analyses (NMAs) has been used to estimate relative efficacy. NMAs extend conventional, pair-wise meta-analysis, and are based on the principle that within trial estimates of relative treatment effects can be added and subtracted [5,6].

An important assumption with NMAs is that the studies used are sufficiently similar in terms of relative treatment effect modifiers [7] - that is, study-level factors that may influence the size of the treatment effect seen with a particular pair-wise intervention. These include patient characteristics, outcomes measured, and study design. Thus, to ensure a fair comparison of interventions, it is essential to control for differences between studies in terms of potential relative treatment effect modifiers. In particular, baseline differences in patient characteristics between different trials may distort between-trial comparisons if appropriate adjustments are not made.

In CHB, response to treatment varies according to the outcome of interest, the agent used, and the patient’s HBeAg status [2]. Patient/disease characteristics that have been shown to predict response to treatment in at least some categories include baseline viral load, serum ALT level, HBV genotype, and activity score on liver biopsy [2,4]. Ali and colleagues [8] analysed data from 1,353 patients in two RCTs of entecavir and found that higher baseline viral load was associated with reduced odds of response to treatment: when baseline viral load (by PCR) was treated as a continuous variable, the odds of achieving a response were reduced by a factor of 0.38 (62%) for every one unit increase in log_10_ PCR above a threshold of 400 copies/ml.

Given the absence of head-to-head RCTs for all interventions, the objective of the current study was to generate estimates of relative efficacy of achieving undetectable viral load (UVL) that take into account the potential of baseline viral load to act as a treatment effect modifier, in order to provide like-for-like comparisons between treatments for CHB that take into account the heterogeneity in baseline viral load across patient populations in different trials.

In order to compare the results with previously published NMAs, as well as demonstrate the implications for clinical and reimbursement decisions of using such estimates, we also generated unadjusted relative efficacy estimates using similar methodologies to those used in these previous analyses.

## Methods

We carried out adjusted and unadjusted analyses, using the same trial data for each, to explore the impact of baseline viral load on treatment response at 1 year. The interventions analysed at licensed doses were interferon alfa, peginterferon alfa-2a/2b, lamivudine, adefovir dipivoxil, entecavir, tenofovir, telbivudine and also placebo. Trials for inclusion in the NMAs were identified through a systematic review of the literature.

The efficacy endpoints analysed in the unadjusted analysis were ALT normalization, histological improvement, HBeAg seroconversion and achievement of UVL at 1 year. Since Ali and colleagues [8] only generated results for one endpoint (achievement of UVL at 1 year) the adjusted analysis was necessarily restricted to this endpoint/timepoint. In all analyses, UVL was defined as reduction in HBV DNA level (by PCR assay) below the trial specific lower level of quantification (LLOQ).

### Systematic review

We carried out a systematic review of RCTs of the interventions listed above. The inclusion criteria were RCTs (phase II or III) of monotherapy interventions at licensed dose, adults with CHB, reporting any of the endpoints of interest, and published in English. Papers (full or otherwise) reporting interim results and studies using the interventions of interest at non-licensed doses were excluded.

Searches were carried out in the Embase, Medline, Medline in Process and Cochrane CENTRAL databases between March and April 2011. No restriction was placed on the earliest date of publication and all databases were searched from date of inception. Search strategies comprised CHB disease and drug terms (a combination of controlled vocabulary and free text terms), and also a bespoke RCT filter. A search was also made for abstracts from the European Association for the Study of the Liver and the American Association for the Study of Liver Diseases 2010 and 2011 annual conferences. Search syntax for all databases are available on request from the authors. The search strategy used to search the Embase database is presented in Additional file 1.

The studies were separated into four clinically distinct patient groups: treatment-naïve HBeAg-positive or -negative, lamivudine refractory and ‘other’. Abstract screening was performed by two authors and included in the full paper review if one reviewer thought it relevant. Formal full paper review was undertaken by two reviewers against the pre-specified inclusion criteria with a third acting as mediator in situations of disagreement. Three authors independently extracted study characteristics and the outcome data required for the NMA using a standard form. Discrepancies were resolved by one of two other authors. Outcome data from weeks 48 and 52 were assumed to refer to 1 year. A risk of bias assessment was carried out using Cochrane methodology for those RCTs reported as full papers [9]. No formal protocol was created for this review.

### Statistical analyses

Given the lack of head-to-head trial evidence estimating the relative efficacy of all licensed interventions, we used an NMA approach to synthesise the evidence. In the NMA methodology the difference in effect between treatments A and B is equal to the difference in effects between treatments A and C, and B and C. The analysis can be expanded to more complex networks of evidence, and can produce estimates of both mean effect and uncertainty [10].

Fixed effect models were used in the unadjusted analysis. For the adjusted analyses we used both fixed and random effects models, and final model choice in all analyses was based on deviance information criteria (DIC) [11].

Choice of prior distribution for parameters in NMA models is an important consideration, especially in the presence of sparse networks of evidence. Uninformative priors were used in the unadjusted analyses for all model parameters. In all covariate-adjusted analyses the results from Ali and colleagues [8] were used to inform the prior distribution on the regression coefficient associated with baseline viral load. Variations in baseline viral load in each study arm (where there were differences) were incorporated into the adjusted analyses via the use of the average baseline HBV DNA value across arms within a given RCT.

Baseline viral load was assumed to modify treatment effects relative to entecavir (0.5 mg), which was also used as the baseline against which all relative efficacy estimates were calculated. To make the results easier to interpret by a non-statistical audience, we represented relative efficacy as a relative risk (RR) of response instead of the more natural odds ratio. We reported the mean of the posterior probability distribution as well as the 95% credible interval (CrI) for each RR. When the 95% CrI did not include the value one, the RR was considered significantly different to that for entecavir.

In order to compare the results of the analyses with the input data, as well as presenting the output in an intuitive manner, we also generated the absolute predicted posterior probabilities of response for each clinical outcome and treatment combination. In the adjusted NMA we also undertook a range of sensitivity analyses whereby in addition to the use of fixed and random effects models, the impact of adding or removing individual studies due to heterogeneity was assessed. Caterpillar, density and Brooks-Gelman-Rubin plots were examined in all analyses to ensure model convergence.

The analyses were conducted in WinBUGS Version 1.4 (MRC Biostatistics Unit, Cambridge, UK) [12] using Bayesian Markov Chain Monte-Carlo Gibbs sampling methods.

## Results

### Search results and summary of studies

Our search of clinical databases identified 3,000 abstracts; 179 articles, including clinical study reports (CSRs), were ordered/requested for review, of which 35 (six CSRs) met the inclusion criteria [13-48]. The contents of five of the CSRs had been reported in peer reviewed publications already captured by the search and hence the published data were used [18,26,37,42,44]. One CSR (BMS study AI463023) [13] and the Summary of Product Characteristics for telbivudine [22] were included in the review. In total, the review identified 29 unique trials. Of these, 19 contained information in HBeAg-positive patients, and 14 of the 19 reported enough information to warrant inclusion in a NMA, and 13 reported information on UVL at that timepoint [13-15,18,20-24,26,28,30,32,48].

**Figure 1:**
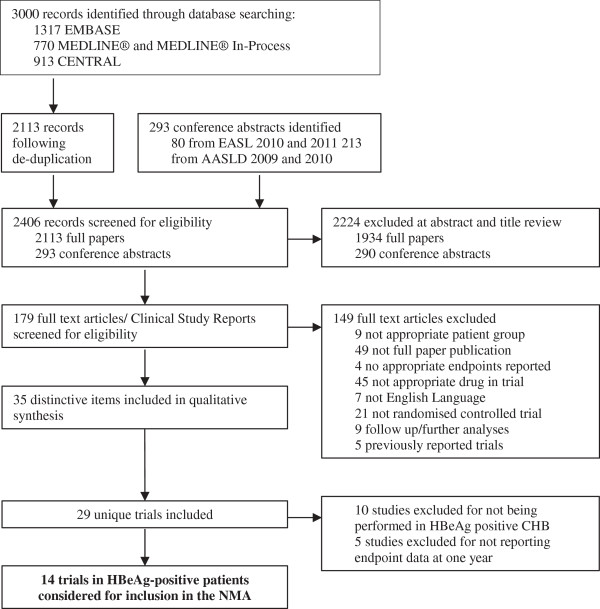
**PRISMA diagram of studies included in the systematic review.** AASLD, American Association for the Study of Liver Diseases; CHB, chronic hepatitis B; EASL, European Association for the Study of the Liver; HBeAg, hepatitis B e antigen; NMA, network meta-analysis.

The study selection process is presented as a PRISMA diagram in Figure 1. The PRISMA 2009 checklist is reportedin Additional file 2. Study characteristics and reported UVL at 1 year (defined as either 48 or 52 weeks) are shown in Table 1. The assessment of study quality undertaken as part of the systematic review is reported in Additional file 1: Table S1. Studies identified by the systematic review used a range of LLOQ values from 1,000 to 200 copies/ml.

**Table 1:** Study characteristics and 1-year outcomes of studies included in the network meta-analysis (HBeAg-positive patients only)

**Source**	**Treatment duration**	**Study design**	**Number of patients**	**Treatment**	**Age (years)**	**Male (%)**	**Endpoint timepoint**	**Method used to measure HBV DNA**	**LLOQ**	**Baseline viral load (log**_**10**_**copies/ml)**	**Undetectable HBV DNA at 1 year (%)**
018 Study Group [13]	52 weeks	Randomised, controlled, open label	45	TBV 600 mg	34	78	52 weeks	Amplicor PCR Assay (Roche)	300 copies/ml	9.57	60
			44	ADV 10 mg	30	91				9.98	40.9
ADV 437 Study Group [14]	48 weeks	Randomised, single blind	167	Placebo	37	71	48 weeks	Amplicor PCR Assay (Roche)	400 copies/ml	8.12	0
			171	ADV 10 mg	34	76				8.25	21.1
AHLSG [15]	52 weeks	Randomised, double blind	72	Placebo	29	72	52 weeks	Solution hybridising assay (Abbott)	1.6 pg/ml	1.85	NR
			143	LAM 100 mg	31	74				1.8	NR
AI463023 [12]	96 weeks	Phase 3 randomised, double blind	225	ETV 0.5 mg	-	-	52 weeks	PCR assay (company unspecified)	300 copies/ml	8.80	73.8
			221	LAM 100 mg	-	-				8.70	37.6
BeHoLD_I [17]	60 weeks	Phase 3 randomised, double blind	354	ETV 0.5 mg	35	77	48 weeks	Amplicor PCR Assay (Roche)	300 copies/ml	9.62	66.7
			355	LAM 100 mg	35	74				9.69	36.3
Globe study group [19-21]	NR	Phase 3 randomised, double blind	463	LAM 100 mg	33	76	52 weeks	Amplicor PCR Assay (Roche)	300 copies/ml	9.50	40.4
			458	TBV 600 mg	32	73				9.50	60
Hou [22]	104 weeks	Phase 3 randomised, double blind	147	TBV 600 mg	28	80	52 weeks	Amplicor PCR Assay (Roche)	300 copies/ml	9.30	66.7
			143	LAM 100 mg	29	75				9.70	37.8
ILSG [23]	52 weeks	Randomised, partially double blind	82	LAM 100 mg	30	71	52 weeks	Solution hybridising assay (Abbott)	1.6 pg/ml	2.04	60
			69	IFNA	32	81				1.78	29.1
Lau [47]	72 weeks	Phase 3 randomised, double blind	271	PegIFNA	32.5	79	48 weeks	Amplicor PCR Assay (Roche)	400 copies/ml	9.90	25.1
			272	LAM 100 mg	31.6	79				10.10	39.7
Leung [25]	Minimum 52 weeks	Phase 3 randomised, open label	33	ETV 0.5 mg	37	61	48 weeks	Amplicor PCR Assay (Roche)	300 copies/ml	10.30	57.6
			32	ADV 10 mg	32	66				9.88	18.8
Marcellin [26]	48 weeks	Phase 3 randomised, double blind	176	TDF 300 mg	34	68	48 weeks	Cobas Taq-Man PCR Assay (Roche)	169 copies/ml	8.64	76.1
			90	ADV 10 mg	34	71				8.88	13.3
Ren [27]	48 weeks	Randomised	21	LAM 100 mg	34	52	48 weeks	PCR assay (company unspecified	Unspecified	8.49	38
			21	ETV 0.5 mg	31	57				8.52	71.4
TBVIG [29]	52 weeks	Phase 2 randomised, double blind	19	LMV 100 mg	34	74	52 weeks	Amplicor PCR Assay (Roche)	200 copies/ml	N/R	31.6
			22	TBV 600 mg	40	82				N/R	61.4
USLIG [30]	68 weeks	Prospective, randomised, double blind	71	Placebo	38	80	52 weeks	Unspecified	Unspecified	5.70	15.9
			66	LAM 100 mg	40	86				10.20	44.4

The systematic review identified 21 studies reporting hepatitis B e antigen (HBeAg)-positive patients; this table only shows the 14 studies included in the network meta-analysis. The remaining 7 studies did not report outcomes at 1 year and so were not included in the network meta-analysis. *Patient numbers for overall population provided, baseline viral load and hepatitis B virus (HBV) DNA reported as HBeAg-positive/HBeAg-negative, outcomes for HBeAg-positive patients only. ADV, adeforvir; ETV, entecavir; IFNA, interferon-alfa; LAM, lamivudine; LLOQ, lower level of quantification; NR, not reported; PegIFNA, pegylated interferon-alfa 2a; TBV, telbivudine; TDF, tenofovir.

### Unadjusted network meta-analysis

**Figure 2:**
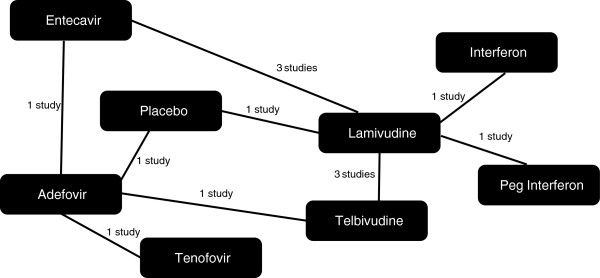
Evidence networks of studies used to generate unadjusted results for the undetectable viral load endpoint.

The network of evidence used to generate all results is presented in Figure 2. The results of the fixed effects analysis are presented as relative risks in Table 2 and absolute probabilities of response in Additional file 1: Table S2. There was only one instance where a treatment performed significantly better than entecavir: the RR for tenofovir achieving UVL was 1.43 (95% CrI 1.30 to 1.54). With the exception of telbivudine which demonstrated no statistically significant difference to entecavir (RR 0.88, 95% CrI 0.76 to 1.00) all other interventions performed significantly less well than entecavir.

**Table 2:** Unadjusted efficacy estimates relative to treatment with entecavir

	**Fixed effects model**
Entecavir 0.5 mg	1.00 (Baseline)
Adefovir 10 mg	0.64 (0.42, 0.87)
Lamivudine 100 mg	0.54 (0.46, 0.63)
Placebo	0.10 (0.04, 0.19)
Telbivudine 600 mg	0.88 (0.76, 1.00)
Tenofovir 245 mg	1.43 (1.30, 1.54)
Interferon alfa	0.21 (0.09, 0.38)
Peginterferon alfa-2a/2b	0.34 (0.23, 0.46)

Results are shown as relative risk (95% credible interval). A relative risk <1 should be interpreted as a given treatment being less efficacious (that is, worse) than entecavir, and a value >1 being more efficacious (that is, better).

### Adjusted network meta-analysis

The primary adjusted analysis of achieving UVL at 1 year, when accounting for baseline viral load, was undertaken using materials available in the public domain (the “base case”). Thus, the material extracted from the CSR was excluded. In addition, the baseline rates for two studies were very different to the remainder in that they were assessed using a different assay with very different LLOQ definitions suggesting that baseline data were collected in a different manner to all other studies [16,24]. These studies were also excluded from the base case analysis. One study, TBVIG, reported median rather than mean baseline viral load and was hence also excluded from the base case-adjusted analysis [30]. Information on this study is provided in Table 1. Data from the ten studies that reported baseline viral load were used in the adjusted analyses [14,15,18,20-23,26-28,31,48].

**Figure 3:**
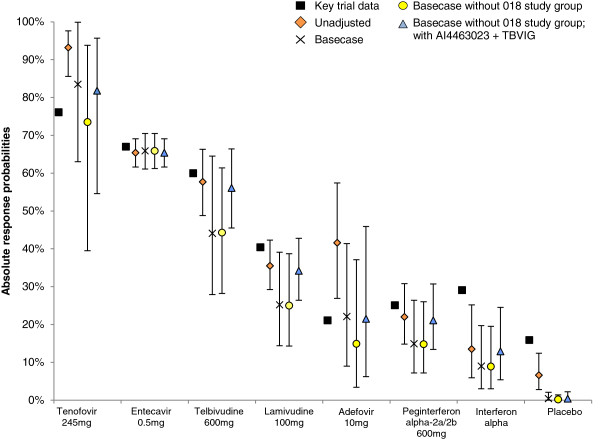
**Absolute probability of undetectable viral load at 1 year (fixed effects).** “Basecase” refers to the adjusted analysis undertaken using the ten studies listed in the document containing appropriate information.

**Figure 4:**
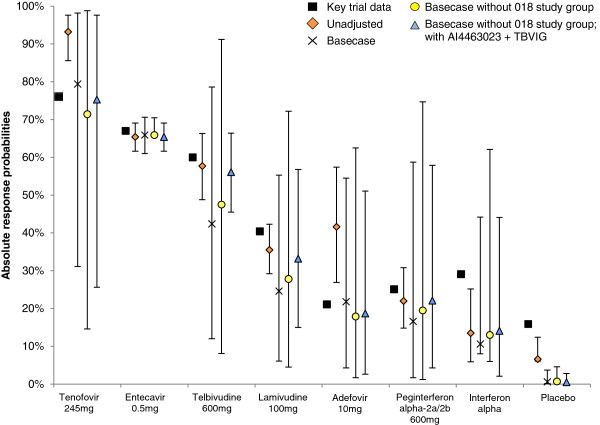
**Absolute probability of undetectable viral load at 1 year (random effects).** “Basecase” refers to the adjusted analysis undertaken using the ten studies listed in the document containing appropriate information.

The results are presented as relative efficacy estimates in the second column of Table 3 and as absolute probabilities of UVL at 1 year in Figure 3 (fixed effects) and Figure 4 (random effects).

**Table 3:** Adjusted relative risk estimates for virologic response, expressed as relative risk of achieving undetectable viral load

**Intervention**	**Base case**	**Base case without 018 study group**	**Base case without 018 study group; with AI4463023 + TBVIG**
**Fixed effects analyses**			
Entecavir 0.5 mg	1.00 (Baseline)	1.00 (Baseline)	1.00 (Baseline)
Adefovir 10 mg	0.33 (0.14, 0.62)	0.23 (0.05, 0.56)	0.33 (0.10, 0.70)
Lamivudine 100 mg	0.38 (0.22, 0.58)	0.38 (0.22, 0.58)	0.52 (0.41, 0.64)
Placebo	0.01 (0.00, 0.03)	0.00 (0.00, 0.02)	0.01 (0.00, 0.03)
Telbivudine 600 mg	0.67 (0.43, 0.92)	0.67 (0.44, 0.92)	0.86 (0.71, 1.01)
Tenofovir 245 mg	1.27 (0.96, 1.47)	1.12 (0.61, 1.43)	1.25 (0.84, 1.48)
Interferon alpha	0.14 (0.05, 0.29)	0.13 (0.05, 0.29)	0.20 (0.08, 0.37)
Peginterferon alpha-2a/2b	0.23 (0.11, 0.39)	0.22 (0.11, 0.39)	0.32 (0.21, 0.46)
Residual deviance	19.48	17.10	21.54
DIC	35.56	32.14	39.15
**Random effects analyses**			
Entecavir 0.5 mg	1.00 (Baseline)	1.00 (Baseline)	1.00 (Baseline)
Adefovir 10 mg	0.33 (0.07, 0.82)	0.27 (0.03, 0.95)	0.29 (0.04, 0.78)
Lamivudine 100 mg	0.37 (0.09, 0.84)	0.42 (0.07, 1.09)	0.51 (0.23, 0.86)
Placebo	0.01 (0.00, 0.06)	0.01 (0.00, 0.07)	0.01 (0.00, 0.04)
Telbivudine 600 mg	0.64 (0.18, 1.19)	0.72 (0.12, 1.39)	0.86 (0.41, 1.27)
Tenofovir 245 mg	1.21 (0.48, 1.51)	1.08 (0.22, 1.52)	1.15 (0.39, 1.50)
Interferon alpha	0.16 (0.01, 0.67)	0.20 (0.01, 0.94)	0.22 (0.03, 0.67)
Peginterferon alpha-2a/2b	0.25 (0.03, 0.88)	0.30 (0.02, 1.13)	0.34 (0.07, 0.88)
Residual deviance	18.23	16.12	19.78
DIC	35.86	32.10	39.07

Results are shown as relative risk (95% credible interval). “Base case” refers to the adjusted analysis undertaken using the 10 studies listed in the document containing appropriate information. As with the unadjusted analyses, a relative risk <1 should be interpreted as a given treatment being less efficacious (that is, worse) than entecavir and a value >1 being more efficacious (that is, better). DIC, deviance information criteria.

The relative risk estimates produced by the fixed and random effects base case analyses were very similar. In particular, entecavir produced significantly increased RRs of UVL at 1 year compared with all interventions except telbivudine and tenofovir, for which the likelihood was similar. In contrast to the unadjusted analysis, the relative efficacy of entecavir and tenofovir for achieving UVL was not significantly different. Thus, baseline viral load is a significant moderator of the effects of monotherapies for CHB. The DIC estimate for the fixed effect model was lower than that for the random effects model and was thus the preferred approach.

### Adjusted network meta-analysis: sensitivity analyses

#### Exclusion of data from one adefovir study

The reported 1 year UVL rate for adefovir patients as reported by the 018 Study Group is approximately two to three times higher than reported for adefovir in all other studies (Table 1). In contrast, the absolute response rate for patients receiving telbivudine reported by this study was in line with that observed in the other studies. The impact of removing this study is presented in column three of Table 3, and in Figures 3 and 4.

The DIC statistics for both fixed and random effects models were similar, with the random effects analysis representing the best fitting model. While the results overall are similar to those in the base case analyses, the greatest impact is observed in the tenofovir results, with a relative risk value of 1.08 (95% CrI 0.22 to 1.52). Of note, the derived absolute response probabilities for entecavir and tenofovir in this scenario were 65.9% and 71.4%, respectively (random effects model). The corresponding values in the key regulatory trials were 66.7% and 76.5%, respectively.

#### Exclusion of data from the 018 Study Group, and inclusion of data from AI463023 and TBVIG

The systematic review identified two additional studies which contained information of potential interest: as yet unpublished data from BMS study AI463023 and median baseline values from the TBVIG study [13,29]. When these data were included, but the data from 018 remained excluded, the corresponding results from this analysis are presented in column four of Table 3, and in Figures 3 and 4.

The random effects analysis generated the lowest DIC and is therefore the preferred model. There was no significant difference between the relative efficacy of entecavir, telbivudine and tenofovir, but entecavir performed significantly better than all other interventions. These results are in contrast to the unadjusted results. The absolute probabilities derived using a random effects model for tenofovir, telbivudine and entecavir were similar to those observed in the landmark RCTs.

## Discussion

NMA can be used to generate relative efficacy estimates of competing treatments in situations where more than two treatment options are available and direct head-to-head evidence from RCTs does not exist for all comparators. The NMA approach allows all relevant evidence to be considered and addresses research questions in the absence of direct comparative evidence, improving the precision of estimates by combining direct and indirect evidence.

One of the key assumptions underpinning this method is that the studies included in the analysis are homogeneous (that is, the trials are sufficiently similar on study and patient characteristics). The similarity assumption is violated if one or more study-level covariates act as modifiers of the relative treatment effects and their distribution is not balanced across the studies being compared [49,50]. In this case, NMA may be affected by confounding bias, unless one explicitly controls for these covariates in the statistical analyses.

Controlling for covariates is particularly important in cases where response to treatment is defined in terms of post-treatment level of a measure, and when that baseline level of this measure is known to vary across studies. If one study recruits patients with worse levels of a variable that is known to modify the relative impact of treatment, then the level of response achieved is likely to be smaller compared with another study which primarily includes patients with better baseline levels, other things being equal.

The motivation for our work was the belief that such baseline covariate imbalances had occurred for patients recruited into studies looking at interventions for CHB. In particular, it was noted that there were differences in mean baseline viral load (expressed in terms of log_10_ copies/ml when measured using the PCR assay) with values for entecavir and tenofovir differing by approximately 1 log_10_ copies/ml (Table 1). We hypothesised that failure to account for these differences in previous analyses may have led to biased estimates of relative efficacy.

The work contained in this paper supports this hypothesis. When no adjustment was made to account for differences in baseline viral load among trials, tenofovir was shown to be significantly better than entecavir in terms of achieving UVL at 1 year (fixed effects RR 1.43, 95% CrI 1.30 to 1.54). However, when we accounted for the impact of baseline viral load the difference between the two treatments was not significant (fixed effects RR 1.27, 95% CrI 0.96 to 1.47; random effects RR 1.21, 95% CrI 0.48 to 1.51). The fixed effects adjusted model best fitted the underlying data, although the difference was minor (fixed effects DIC, 35.56; random effects DIC, 35.86).

Sensitivity analyses highlighted that the relative efficacy of tenofovir versus entecavir was contingent on the choice of studies included in the meta-analysis, and in particular whether or not data reported by one study group [13] were used. When these data were excluded, there is no significant difference between the two interventions (RR 1.08, 95% CrI 0.22 to 1.52). A subsequent sensitivity analysis, whereby this study was removed but two other studies were included (AI463023 and TBVIG), generated similar non-significant results (RR 1.15, 95% CrI 0.39 to 1.50). In both sensitivity analyses the most appropriate model, based on DIC, consisted of random as opposed to fixed effects approaches. Close examination of the published paper [14] has identified no reason why this result should occur, and so there may be some other form of study level heterogeneity as yet unaccounted for that is influencing the results.

Our paper is the first to generate baseline viral load adjusted and unadjusted NMA results using data from the same set of studies, and the results from the unadjusted analyses are very similar to those generated by other research groups [51,52]. Accepting that NMA is based on relative efficacy, the results from all three unadjusted analyses for UVL appear to be at odds with those provided by the clinical trials included in the NMA. The systematic review identified one study of tenofovir [27] and the observed response rate was 76%. The corresponding value arising from our NMA was 93.2% (95% CrI 85.6% to 97.6%). Similar values were generated by two other research groups [51,52]. One other NMA has been recently published [53]. This analysis, however, contains a number of methodological flaws, the most notable being the pooling of data from HBeAg-positive and -negative individuals. We have therefore not extracted results from this paper for the purposes of discussion.

In contrast, with the exception of placebo and interferon-based therapies, the CrIs for the values derived in the adjusted analyses all contain the observed trial values, and the RR estimates are close to the trial values once the 018 Study Group data are removed (Figures 3 and 4). Hence, we would argue that the adjusted results are of greater clinical relevance than the unadjusted results.

Generating ‘like-for-like’ estimates of relative efficacy by controlling for covariates believed to be modifiers of relative treatment effects is not just of clinical interest but is essential for the purposes of reimbursement decisions. Such estimates are used by agencies such as the National Institute for Health and Care Excellence in their appraisal processes when assessing the clinical efficacy in a given disease area [54]. In addition, such values are also used in economic models to evaluate the cost-effectiveness of interventions. A number of such models have been developed in CHB [55-59], of which one [57] used the results from their unadjusted analysis directly as model inputs. Another [59] used UVL as a surrogate variable for risk of cirrhosis using information from the REVEAL-HBV study [60], which quantified the relationship between HBV DNA and the likelihood of being diagnosed with cirrhosis. Overestimation of virologic response would thus correspond to underestimation of the likelihood of cirrhosis, which has been identified as a key driver of cost-effectiveness.

Despite the review finding a decent number of studies overall, as can be seen from Figure 2, the presence of a large number of treatment options means that the majority of the branches in the network are informed by the findings of a single study. This increases the uncertainty surrounding all results and means that baseline imbalances in other potential treatment effect modifiers may have influenced the results.

Further work is needed to complement the work contained in this paper in connection with the achievement of UVL at 1 year in order to explore the impact of other potentially clinically relevant covariates on the relative effects of comparators and the probability of achieving UVL. Exploring the impact of other areas of potential heterogeneity (for example, study design, impact of different LLOQ definitions) is also important. In addition, Ali and collagues [8] identified the time of assessment as a treatment effect modifier in addition to baseline viral load. The studies included in this analysis were very similar in terms of assessment times and so the exclusion of this variable is likely to have had a modest effect. Nonetheless, it would be interesting to replicate the analyses contained in this paper when controlling for these slight differences. Furthermore, expanding this type of analysis to other clinically relevant endpoints is also worthwhile.

## Conclusions

The analysis showed that baseline viral load is a treatment effect modifier in CHB and that failure to correct for this variable inflates the relative efficacy estimates for some interventions. Since these estimates are often used in economic models to generate cost-effectiveness estimates, failure to adjust for baseline viral load will generate erroneous ICERs, resulting in poor use of scarce healthcare resources. As such, reimbursement agencies should therefore only use covariate-adjusted relative efficacy estimates in their decision making surrounding treatments for CHB.

## Abbreviations

ALT: alanine transaminase; CHB: chronic hepatitis B; CrI: credible interval; CSR: clinical study report; DIC: deviance information criteria; HBeAg: hepatitis B e antigen; HBV: hepatitis B virus; HCC: hepatocellular carcinoma; LLOQ: lower level of quantification; NMA: network meta-analysis; PCR: polymerase chain reaction; RCT: randomised clinical trials; RR: relative risk; UVL: undetectable viral load.

## Competing interests

SM, MW, NH and DAS all work for an international consultancy and have received funding from numerous pharmaceutical companies including Bristol-Myers Squibb. BL and IG are independent consultants and have received funding from numerous pharmaceutical companies including Bristol-Myers Squibb. EM and BB are employees of Bristol-Myers Squibb. PL, MT, LM and MC have all received speaking bureau (paid speaking invitation) from multiple pharmaceutical companies including Bristol-Myers Squibb.

## Authors’ contributions

IG performed and validated all network meta-analyses. NH and DAS performed and validated all network meta-analyses and also acted as adjudicators in the systematic review process as well as acting as co-supervisors for the project. SM assisted in all aspects of the systematic review, led the preparation of the manuscript, worked with SA in developing the patient level data analysis and performed day-to-day management of the project. MW designed all search strategies used in the systematic review and performed additional systematic review tasks including checking data extraction. BB and EM obtained funding for the project, contributed to the project analysis protocol (unpublished) and acted as external clinical reviewers to the process. As such the authors performed technical and clinical validation on all inputs and outputs to the process. BL contributed to the project analysis protocol (unpublished) and acted as external clinical reviewer to the process. As such the author performed technical and clinical validation on all inputs and outputs to the process, and was also responsible for developing the study concept. MT, PL, LM and MC contributed to the project analysis protocol (unpublished) and acted as external clinical reviewers to the process. As such the authors performed technical and clinical validation on all inputs and outputs to the process. All authors read and approved the final manuscript.

## Supplementary Material

Additional file 1 Supplementary material, including Embase search strategy, assessment of study quality for all included trials (Table S1) and comparison of current and previous NMA results (Table S2). Click here for file

Additional file 2 PRISMA 2009 checklist. Click here for file

## References

[B1] World Health Organization Hepatitis B: Fact Sheet 204 http://who.int/mediacentre/factsheets/fs204/en/

[B2] European Association for the Study of the Liver EASL clinical practice guidelines: management of chronic hepatitis B virus infection J Hepatol 2012 57 167 185 22436845 10.1016/j.jhep.2012.02.010

[B3] Bosch FX Ribes J Cléries R Díaz M Epidemiology of hepatocellular carcinoma Clin Liver Dis 2005 9 191 211 10.1016/j.cld.2004.12.009 15831268

[B4] Lok ASF McMahon BJ AASLD practice guidelines. Chronic hepatitis B: update 2009 Hepatology 2009 50 661 662 10.1002/hep.23190 19714720

[B5] Caldwell D Ades AE Higgins JP Simultaneous comparison of multiple treatments: combining direct and indirect evidence BMJ 2005 331 897 900 10.1136/bmj.331.7521.897 16223826 PMC1255806

[B6] Lu G Ades AE Combination of direct and indirect evidence in mixed treatment comparisons Stat Med 2004 23 3105 3124 10.1002/sim.1875 15449338

[B7] Song F Loke YK Walsh T Glenny AM Eastwood AJ Altman DG Methodological problems in the use of indirect comparisons for evaluating healthcare interventions: survey of published systematic reviews BMJ 2009 338 b1147 10.1136/bmj.b1147 19346285 PMC2665205

[B8] Ali S Mealing S Hawkins N Lescrauwaet B Bjork S Mantovani L Lampertico P The use of individual patient data (IPD) to quantify the impact of pre-treatment predictors of response to treatment in chronic hepatitis B patients BMJ Open 2013 24 3 10.1136/bmjopen-2012-001309 PMC3563125 23355658

[B9] Cochrane Handbook for Systematic Reviews of Interventions Version 5.0.2 http://www.cochrane.org/training/cochrane-handbook

[B10] Hoaglin DC Hawkins N Jansen JP Scott DA Itzler R Cappelleri JC Boersma C Thompson D Larholt KM Diaz M Barrett A Conducting indirect-treatment-comparison and network-meta-analysis studies: report of the ISPOR Task Force on Indirect Treatment Comparisons Good Research Practices: part 2 Value Health 2011 14 429 437 10.1016/j.jval.2011.01.011 21669367

[B11] Spiegelhalter DJ Best NG Carlin BP Van Der Linde AJ Bayesian measures of model complexity and fit Royal Stat Soc: Series B (Stat Methodol) 2002 64 583 639 10.1111/1467-9868.00353

[B12] Lunn DJ Thomas A Best N Spiegelhalter D WinBUGS – a Bayesian modelling framework: concepts, structure, and extensibility Stat Comput 2000 10 325 337 10.1023/A:1008929526011

[B13] Bristol-Myers Squibb Ltd Trial AI463023 Clinical Study Report Data on file

[B14] Chan HL Heathcote EJ Marcellin P Lai CL Cho M Moon YM Chao YC Myers RP Minuk GY Jeffers L Sievert W Bzowej N Harb G Kaiser R Qiao XJ Brown NA 018 Study Group Treatment of hepatitis B e antigen positive chronic hepatitis with telbivudine or adefovir: a randomized trial Ann Intern Med 2007 147 745 754 10.7326/0003-4819-147-11-200712040-00183 17909201

[B15] Marcellin P Chang TT Lim SG Tong MJ Sievert W Shiffman ML Jeffers L Goodman Z Wulfsohn MS Xiong S Fry J Brosgart CL Adefovir Dipivoxil 437 Study Group Adefovir dipivoxil for the treatment of hepatitis B e antigen-positive chronic hepatitis B N Engl J Med 2003 348 808 816 10.1056/NEJMoa020681 12606735

[B16] Lai CL Chien RN Leung NW Chang TT Guan R Tai DI Ng KY Wu PC Dent JC Barber J Stephenson SL Gray DF A one-year trial of lamivudine for chronic hepatitis B N Engl J Med 1998 339 61 68 10.1056/NEJM199807093390201 9654535

[B17] Bristol-Myers Squibb Ltd Trial AI463022 Clinical Study Report Data on file

[B18] Chang TT Gish RG de Man R Gadano A Sollano J Chao YC Lok AS Han KH Goodman Z Zhu J Cross A DeHertogh D Wilber R Colonno R Apelian D BEHoLD AI463022 Study Group A comparison of entecavir and lamivudine for HBeAg-positive chronic hepatitis B N Engl J Med 2006 354 1001 1010 10.1056/NEJMoa051285 16525137

[B19] Cooksley WG Piratvisuth T Lee SD Mahachai V Chao YC Tanwandee T Chutaputti A Chang WY Zahm FE Pluck N Peginterferon alpha-2a (40 kDa): an advance in the treatment of hepatitis B e antigen-positive chronic hepatitis B J Viral Hepat 2003 10 298 305 10.1046/j.1365-2893.2003.00450.x 12823597

[B20] Liaw YF Gane E Leung N Zeuzem S Wang Y Lai CL Heathcote EJ Manns M Bzowej N Niu J Han SH Hwang SG Cakaloglu Y Tong MJ Papatheodoridis G Chen Y Brown NA Albanis E Galil K Naoumov NV GLOBE Study Group 2-Year GLOBE trial results: telbivudine is superior to lamivudine in patients with chronic hepatitis B Gastroenterology 2009 136 486 495 10.1053/j.gastro.2008.10.026 19027013

[B21] Lai CL Gane E Liaw YF Hsu CW Thongsawat S Wang Y Chen Y Heathcote EJ Rasenack J Bzowej N Naoumov NV Di Bisceglie AM Zeuzem S Moon YM Goodman Z Chao G Constance BF Brown NA Globe Study Group Telbivudine versus lamivudine in patients with chronic hepatitis B N Engl J Med 2007 357 2576 2588 10.1056/NEJMoa066422 18094378

[B22] Telbivudine Summary of Product Characteristics http://www.ema.europa.eu/docs/en_GB/document_library/EPAR_-_Product_Information/human/000713/WC500049337.pdf

[B23] Hou J Yin YK Xu D Tan D Niu J Zhou X Wang Y Zhu L He Y Ren H Wan M Chen C Wu S Chen Y Xu J Wang Q Wei L Chao G Constance BF Harb G Brown NA Jia J Telbivudine versus lamivudine in Chinese patients with chronic hepatitis B: results at 1 year of a randomized, double-blind trial Hepatology 2008 47 447 454 18080339 10.1002/hep.22075

[B24] Schalm SW Heathcote J Cianciara J Farrell G Sherman M Willems B Dhillon A Moorat A Barber J Gray DF Lamivudine and alpha interferon combination treatment of patients with chronic hepatitis B infection: a randomised trial Gut 2000 46 562 568 10.1136/gut.46.4.562 10716688 PMC1727894

[B25] Bristol-Myers Squibb Ltd Trial AI463079 Clinical Study Report Data on file

[B26] Leung N Peng CY Hann HW Sollano J Lao-Tan J Hsu CW Lesmana L Yuen MF Jeffers L Sherman M Min A Mencarini K Diva U Cross A Wilber R Lopez-Talavera J Early hepatitis B virus DNA reduction in hepatitis B e antigen-positive patients with chronic hepatitis B: a randomized international study of entecavir versus adefovir Hepatology 2009 49 72 79 10.1002/hep.22658 19065670

[B27] Marcellin P Heathcote EJ Buti M Gane E de Man RA Krastev Z Germanidis G Lee SS Flisiak R Kaita K Manns M Kotzev I Tchernev K Buggisch P Weilert F Kurdas OO Shiffman ML Trinh H Washington MK Sorbel J Anderson J Snow-Lampart A Mondou E Quinn J Rousseau F Tenofovir disoproxil fumarate versus adefovir dipivoxil for chronic hepatitis B N Engl J Med 2008 359 2442 2455 10.1056/NEJMoa0802878 19052126

[B28] Ren FY Piao DM Piao XX A one-year trial of entecavir treatment in patients with HBeAg-positive chronic hepatitis B World J Gastroenterol 2007 13 4264 4267 17696259 10.3748/wjg.v13.i31.4264 PMC4250629

[B29] Shindo M Chayama K Mochida S Toyota J Tomita E Kumada H Yokosuka O Sata M Hayashi N Suzuki K Okanoue T Tsubouchi H Ishikawa H Seriu T Omata M Antiviral activity, dose–response relationship, and safety of entecavir following 24-week oral dosing in nucleoside-naive Japanese adult patients with chronic hepatitis B: a randomized, double-blind, phase II clinical trial Hepatol Int 2009 3 445 452 10.1007/s12072-009-9135-0 19669249 PMC2748381

[B30] Lai CL Leung N Teo EK Tong M Wong F Hann HW Han S Poynard T Myers M Chao G Lloyd D Brown NA Telbivudine Phase II Investigator Group A 1-year trial of telbivudine, lamivudine, and the combination in patients with hepatitis B e antigen-positive chronic hepatitis B Gastroenterology 2005 129 528 536 16083710 10.1016/j.gastro.2005.05.053

[B31] Dienstag JL Schiff ER Wright TL Perrillo RP Hann HW Goodman Z Crowther L Condreay LD Woessner M Rubin M Brown NA Lamivudine as initial treatment for chronic hepatitis B in the United States N Engl J Med 1999 341 1256 1263 10.1056/NEJM199910213411702 10528035

[B32] Yao G Wang B Cui Z Yao J Zeng M A randomized double-blind placebo-controlled study of lamivudine in the treatment of patients with chronic hepatitis B virus infection Chin Med J (Engl) 1999 112 387 391 11593504

[B33] Zeng M Mao Y Yao G Wang H Hou J Wang Y Ji BN Chang CN Barker KF A double-blind randomized trial of adefovir dipivoxil in Chinese subjects with HBeAg-positive chronic hepatitis B Hepatology 2006 44 108 116 10.1002/hep.21225 16799983

[B34] Zheng MH Shi KQ Dai ZJ Ye C Chen YP A 24-week, parallel-group, open-label, randomized clinical trial comparing the early antiviral efficacy of telbivudine and entecavir in the treatment of hepatitis B e antigen-positive chronic hepatitis B virus infection in adult Chinese patients Clin Ther 2010 32 649 658 10.1016/j.clinthera.2010.04.001 20435234

[B35] Hadziyannis SJ Tassopoulos NC Heathcote EJ Chang TT Kitis G Rizzetto M Marcellin P Lim SG Goodman Z Wulfsohn MS Xiong S Fry J Brosgart CL Adefovir Dipivoxil 438 Study Group Adefovir dipivoxil for the treatment of hepatitis B e antigen-negative chronic hepatitis B. [Erratum appears in N Engl J Med. 2003 Mar 20;348(12):1192] N Engl J Med 2003 348 800 807 10.1056/NEJMoa021812 12606734

[B36] Bonino F Lau G Marcellin P Hadziyannis S Kitis G Jin R The first detailed analysis of predictors of response in HBeAg-negative chronic hepatitis B: data from a multicenter, randomized, partially double-blind study of peginterferon alfa-2a (40KD) (PEGASYS) alone or in combination with lamivudine vs lamivudine alone Hepatology 2004 40 659A

[B37] Lai CL Shouval D Lok AS Chang TT Cheinquer H Goodman Z DeHertogh D Wilber R Zink RC Cross A Colonno R Fernandes L BEHoLD AI463027 Study Group Entecavir versus lamivudine for patients with HBeAg-negative chronic hepatitis B N Engl J Med 2006 354 1011 1020 10.1056/NEJMoa051287 16525138

[B38] Chan HL Wang H Niu J Chim AM Sung JJ Two-year lamivudine treatment for hepatitis B e antigen-negative chronic hepatitis B: a double-blind, placebo-controlled trial Antiviral Therapy 2007 12 345 353 17591024

[B39] Tassopoulos NC Volpes R Pastore G Heathcote J Buti M Goldin RD Hawley S Barber J Condreay L Gray DF Efficacy of lamivudine in patients with hepatitis B e antigen-negative/hepatitis B virus DNA-positive (precore mutant) chronic hepatitis B Hepatology 1999 29 889 896 10.1002/hep.510290321 10051494

[B40] Marcellin P Lau GK Bonino F Farci P Hadziyannis S Jin R Lu ZM Piratvisuth T Germanidis G Yurdaydin C Diago M Gurel S Lai MY Button P Pluck N Peginterferon Alfa-2a HBeAg-Negative Chronic Hepatitis B Study Group Peginterferon alfa-2a alone, lamivudine alone, and the two in combination in patients with HBeAg-negative chronic hepatitis B N Engl J Med 2004 351 1206 1217 10.1056/NEJMoa040431 15371578

[B41] Bristol-Myers Squibb Ltd Trial AI463026 Clinical Study Report Data on file

[B42] Sherman M Yurdaydin C Sollano J Silva M Liaw YF Cianciara J Boron-Kaczmarska A Martin P Goodman Z Colonno R Cross A Denisky G Kreter B Hindes R AI463026 BEHoLD Study Group Entecavir for treatment of lamivudine-refractory, HBeAg-positive chronic hepatitis B Gastroenterology 2006 2006 2039 2049 16762627 10.1053/j.gastro.2006.04.007

[B43] Bristol-Myers Squibb Ltd Trial AI463014 Clinical Study Report Data on file

[B44] Chang TT Gish RG Hadziyannis SJ Cianciara J Rizzetto M Schiff ER Pastore G Bacon BR Poynard T Joshi S Klesczewski KS Thiry A Rose RE Colonno RJ Hindes RG BEHoLD Study Group A dose-ranging study of the efficacy and tolerability of entecavir in lamivudine-refractory chronic hepatitis B patients Gastroenterology 2005 129 1198 1209 10.1053/j.gastro.2005.06.055 16230074

[B45] Liaw YF Sheen IS Lee CM Akarca US Papatheodoridis GV Suet-Hing Wong F Chang TT Horban A Wang C Kwan P Buti M Prieto M Berg T Kitrinos K Peschell K Mondou E Frederick D Rousseau F Schiff ER Tenofovir disoproxil fumarate (TDF), emtricitabine/TDF, and entecavir in patients with decompensated chronic hepatitis B liver disease Hepatology 2011 53 62 72 10.1002/hep.23952 21254162

[B46] Liaw YF Sung JJ Chow WC Farrell G Lee CZ Yuen H Tanwandee T Tao QM Shue K Keene ON Dixon JS Gray DF Sabbat J Cirrhosis Asian Lamivudine Multicentre Study Group Lamivudine for patients with chronic hepatitis B and advanced liver disease N Engl J Med 2004 351 1521 1531 10.1056/NEJMoa033364 15470215

[B47] Lai CL Rosmawati M Lao J Van Vlierberghe H Anderson FH Thomas N Dehertogh D Entecavir is superior to lamivudine in reducing hepatitis B virus DNA in patients with chronic hepatitis B infection Gastroenterology 2002 123 1831 1838 10.1053/gast.2002.37058 12454840

[B48] Lau GK Piratvisuth T Luo KX Marcellin P Thongsawat S Cooksley G Gane E Fried MW Chow WC Paik SW Chang WY Berg T Flisiak R McCloud P Pluck N Peginterferon Alfa-2a HBeAg-Positive Chronic Hepatitis B Study Group Peginterferon alfa-2a, lamivudine, and the combination for HBeAg-positive chronic hepatitis B N Engl J Med 2005 352 2682 2695 10.1056/NEJMoa043470 15987917

[B49] Jansen JP Naci H Is network meta-analysis as valid as standard pairwise meta-analysis? It all depends on the distribution of effect modifiers BMC Med 2013 11 159 10.1186/1741-7015-11-159 23826681 PMC3707819

[B50] Salanti G Indirect and mixed-treatment comparison, network, or multiple-treatments meta-analysis: many names, many benefits, many concerns for the next generation evidence synthesis tool Res Synth Method 2012 3 80 97 10.1002/jrsm.1037 26062083

[B51] Dakin H Fidler C Harper C Mixed treatment comparison meta-analysis evaluating the relative efficacy of nucleos(t)ides for treatment of nucleos(t)ide-naive patients with chronic hepatitis B Value Health 2010 13 934 945 10.1111/j.1524-4733.2010.00777.x 20825624

[B52] Woo G Tomlinson G Nishikawa Y Kowgier M Sherman M Wong DK Pham B Ungar WJ Einarson TR Heathcote EJ Krahn M Tenofovir and entecavir are the most effective antiviral agents for chronic hepatitis B: a systematic review and Bayesian meta-analyses Gastroenterology 2010 139 1218 1229 10.1053/j.gastro.2010.06.042 20600036

[B53] Wiens A Lenzi L Venson R Correr CC Inajara I Pedroso ML Pontarolo R Comparative efficacy of oral nucleoside or nucleotide analog monotherapy used in chronic hepatitis B: a mixed-treatment comparison meta-analysis Pharmacotherapy 2013 33 144 151 10.1002/phar.1188 23359454

[B54] National Institute for Health and Clinical Excellence Guideline to the Methods of Technology Appraisal 2012 http://www.nice.org.uk/aboutnice/howwework/devnicetech/guidetothemethodsoftechnologyappraisal.jsp

[B55] Veenstra DL Sullivan SD Clarke L Iloeje UH Tafesse E Di Bisceglie A Kowdley KV Gish RG Cost effectiveness of entecavir versus lamivudine with adefovir salvage in HBeAg-positive chronic hepatitis B Pharmacoeconom 2007 25 963 977 10.2165/00019053-200725110-00006 17960954

[B56] Veenstra DL Sullivan SD Dusheiko GM Jacobs M Aledort JE Lewis G Patel KK Cost-effectiveness of peginterferon alpha-2a compared with lamivudine treatment in patients with HBe-antigen-positive chronic hepatitis B in the United Kingdom European J Gastroenterol Hepatol 2007 19 631 638 10.1097/MEG.0b013e3281108079 17625431

[B57] Dakin H Bentley A Dusheiko G Cost-utility analysis of tenofovir disoproxil fumarate in the treatment of chronic hepatitis B Value Health 2010 13 922 933 10.1111/j.1524-4733.2010.00782.x 20825619

[B58] Shepherd J Gospodarevskaya E Frampton G Cooper K Entecavir for the treatment of chronic hepatitis B infection Health Technol Assess 2009 13 Suppl 3 31 36 10.3310/hta13suppl3/05 19846026

[B59] Bristol-Myers Squibb Ltd Entecavir (Baraclude) for the Treatment of Chronic Hepatitis B: Single Technology Appraisal Submission to the National Institute for Health and Clinical Excellence http://www.nice.org.uk/nicemedia/live/11833/40303/40303.pdf

[B60] Iloeje UH Yang HI Su J Jen CL You SL Chen CJ Risk Evaluation of Viral Load Elevation and Associated Liver Disease/Cancer-In HBV (the REVEAL-HBV) Study Group Predicting cirrhosis risk based on the level of circulating hepatitis B viral load Gastroenterology 2006 130 678 686 10.1053/j.gastro.2005.11.016 16530509

