# Supplementary material for: The importance of baseline viral load when assessing relative efficacy in treatment-naïve HBeAg-positive chronic hepatitis B: a systematic review and network meta-analysis
Source: Syst Rev. 2014 Mar 7;3:21. doi: 10.1186/2046-4053-3-21 (PMC4015714; doi:10.1186/2046-4053-3-21)
Supplement: Additional file 1 — Supplementary material, including Embase search strategy, assessment of study quality for all included trials (Table S1) and comparison of current and previous NMA results (Table S2). [file 2046-4053-3-21-S1.docx]

# Supplementary material

## Embase search Strategy:

1 exp Hepatitis B/ (43509)

2 chronic hepatitis B.tw. (9314)

3 (chronic adj3 hepatitis B).tw. (10299)

4 1 or 2 or 3 (44869)

5 interferon alfa-2a.tw. (545)

6 (Roferon adj2 A).tw. (1346)

7 Roferon-A.tw. (1345)

8 interferon alfa2a.tw. (10)

9 (interferon alfa adj 2 2a).tw. (0)

10 (interferon alfa adj2 2a).tw. (548)

11 Interferon ALPHA-2B.tw. (1753)

12 (Interferon ALPHA adj2 2B).tw. (1764)

13 Intron-A.tw. (2947)

14 IntronA.tw. (226)

15 peginterferon alfa-2a.tw. (434)

16 (peginterferon alfa adj2 2a).tw. (436)

17 Pegasys.tw. (1291)

18 Pegasys.tw. (1291)

19 ViraferonPeg.tw. (42)

20 (Viraferon adj2 Peg).tw. (34)

21 lamivudine.tw. (6580)

22 Epivir.tw. (1207)

23 Zeffix.tw. (247)

24 adefovir dipivoxil.tw. (715)

25 Hepsera.tw. (310)

26 entecavir.tw. (918)

27 baraclude.tw. (187)

28 tenofovir.tw. (2188)

29 Viread.tw. (743)

30 telbivudine.tw. (370)

31 Sebivo.tw. (68)

32 or/5-31 (16709)

33 4 and 32 (4953)

34 Clinical trial/ (778062)

35 Randomized controlled trial/ (276405)

36 Randomization/ (50493)

37 Single blind procedure/ (14060)

38 Double blind procedure/ (93981)

39 Crossover procedure/ (30479)

40 Placebo/ (143949)

41 Randomi?ed control* trial*.tw. (61955)

42 Rct.tw. (6544)

43 random*.tw. (591431)

44 Random* allocation.tw. (1153)

45 Random* allocat*.tw. (15251)

46 Allocat* random*.tw. (1587)

47 (allocat* adj2 random*).tw. (17693)

48 Single blind$.tw. (9679)

49 Double blind$.tw. (98014)

50 ((treble or triple) adj blind$).tw. (205)

51 Placebo$.tw. (139095)

52 Prospective study/ (159518)

53 or/34-52 (1298561)

54 Case study/ (9337)

55 Case report.tw. (161832)

56 Abstract report/ or letter/ (605417)

57 or/54-56 (773042)

58 53 not 57 (1268156)

59 33 and 58 (1643)

60 limit 59 to (human and english language) (1301)

## Assessment of study quality

An assessment of the quality of each study is presented in **Table S1**. The following key questions, derived from the Cochrane Handbook for systematic reviews of interventions^9^, formed the basis of this assessment:

Question 1: Was the method used to randomize patients described?

Question 2: Was the method used for allocation concealment described?

Question 3: Were the detailed inclusion/exclusion criteria described?

Question 4: Was the method used to ensure treatment blinding described?

Question 5: Were the patient baseline characteristics described?

Question 6: Were all analyses carried out using data from the Intention To Treat (ITT) patient group?

The Cochrane method was used to evaluate the study quality because the Cochrane method analyses six areas where bias may occur in a trial.

Table S1: Assessment of study quality for all included trials

| **Study** | **Question 1** | **Question 2** | **Question 3** | **Question 4** | **Question 5** | **Question 6** |
| --- | --- | --- | --- | --- | --- | --- |
| 018 Study Group | Yes | Yes | Yes | No | Yes | Yes |
| ADV 437 Study Group | Yes | Yes | Yes | Yes | Yes | Yes |
| AHLSG | No | No | Yes | No | Yes | Yes |
| AI463023 | Yes | No | Yes | Yes | No | Yes |
| BEHoLD_I | Yes | Yes | Yes | Yes | Yes | Yes |
| Globe study group | Yes | No | Yes | No | Yes | Yes |
| Hou2008 | Yes | Yes | Yes | No | Yes | Yes |
| ILSG | Yes | Yes | Yes | No | Yes | Yes |
| Lau2005 | Yes | No | Yes | No | Yes | Yes |
| Leung2009 | No | No | Yes | No | Yes | Yes |
| Marcellin2008 | Yes | Yes | Yes | No | Yes | Yes |
| Ren2007 | No | No | Yes | No | Yes | No |
| TBVIG | Yes | Yes | Yes | No | Yes | Yes |
| USLIG | No | No | Yes | Yes | Yes | Yes |

Table S2: Comparison of current and previous NMA results (unadjusted analyses, HBeAg-positive) NB this is absolute probabilities (95% CrI)

|  |  | **Dakin et al.** | **Woo et al.** | **Current study** |
| --- | --- | --- | --- | --- |
|  | Entecavir | 73.0% (57.3%, 87.7%) | 61.0% (36.0%, 80.0%) | 65.4% (61.6%, 69.1%) |
|  | Lamivudine | 38.1% (33.7%, 42.4%) | 31.0% (16.0%, 48.0%) | 35.5% (29.2%, 42.3%) |
|  | Adefovir | 48.9% (25.8%, 77.1%) | 33.0% (11.0%, 64.0%) | 41.6% (26.9%, 57.4%) |
|  | Placebo | 7.2% (1.6%, 18.7%) | 1.0% (0.0%, 4.0%) | 6.6% (2.8%, 12.4%) |
|  | Telbivudine | 62.5% (44.2%, 81.1%) | 51.0% (30.0%, 70.0%) | 57.7% (48.8%, 66.3%) |
|  | Tenofovir | 93.8% (79.9%, 99.3%) | 88.0% (69.0%, 97.0%) | 93.2% (85.6%, 97.6%) |

## WinBUGS code used to generate results

model

{

beta[1]<-0

logit(txProb[1]) <- alphaMean

rr[1]<-1

or[1]<-exp(0)

#prior on tx effect mean

for (qq in 2:nTx)

{

beta[qq]~dnorm(0.0,0.01)

logit(txProb[qq]) <- alphaMean+beta[qq]

rr[qq] <- txProb[qq]/txProb[1]

or[qq] <- exp(beta[qq])

}

alphaMean ~ dnorm(0,0.001)

# ranking and probability treatment is best

for (qq in 1:nTx)

{

rk[qq] <- (nTx + 1) - rank(rr[],qq)

ProbBest[qq] <- equals(rank(rr[ ],qq), nTx)

}

#define prior on intercept

#random baseline effect

for (s in 1:nStudies)

{

alpha[s] ~ dnorm(0,0.001)

}

bCov ~ dnorm(0.59,11.1) # informed prior on covariate coeff

bvl_bar <- 8.72 # centres covariate

#fit data

for(bb in 1:baseNObs ) {

#logit link for probability of response control and treatment arms

logit(baseProb[bb]) <- alphaMean

#binomial link between number of responses and

# probability of response from treatment arm

baseR[bb] ~ dbin(baseProb[bb], baseN[bb])

#resid[ii] <- (r[ii] - n[ii] * cumFail[ii]) / sqrt(n[ii]*cumFail[ii]*(1-cumFail[ii]))

}

#fit data

for(ii in 1:nObs ) {

logOdds[ii] <- alpha[study[ii]] + beta[tx[ii]] - beta[base[ii]]

+ bCov*(bvl[ii] - bvl_bar)*(1-equals(tx[ii],1))

#logit link for probability of response control and treatment arms

logit(prob[ii]) <- logOdds[ii]

r[ii] ~ dbin(prob[ii], n[ii])

rhat[ii] <- prob[ii]*n[ii]

dev[ii] <- 2*( r[ii]*( log(r[ii])-log(rhat[ii]) )

+ (n[ii] - r[ii])*( log(n[ii]-r[ii]) - log(n[ii]-rhat[ii]) ) )

#resid[ii] <- (r[ii] - n[ii] * cumFail[ii]) / sqrt(n[ii]*cumFail[ii]*(1-cumFail[ii]))

}

resdev <- sum(dev[])

}

# Data

list(study=c(1.00000E+00, 1.00000E+00, 2.00000E+00, 2.00000E+00, 3.00000E+00, 3.00000E+00, 4.00000E+00, 4.00000E+00, 5.00000E+00, 5.00000E+00, 6.00000E+00, 6.00000E+00, 7.00000E+00, 7.00000E+00, 8.00000E+00, 8.00000E+00, 9.00000E+00, 9.00000E+00), base=c(2.00000E+00, 2.00000E+00, 1.00000E+00, 1.00000E+00, 3.00000E+00, 3.00000E+00, 3.00000E+00, 3.00000E+00, 3.00000E+00, 3.00000E+00, 1.00000E+00, 1.00000E+00, 2.00000E+00, 2.00000E+00, 2.00000E+00, 2.00000E+00, 3.00000E+00, 3.00000E+00), tx=c(5.00000E+00, 2.00000E+00, 1.00000E+00, 3.00000E+00, 5.00000E+00, 3.00000E+00, 3.00000E+00, 5.00000E+00, 8.00000E+00, 3.00000E+00, 1.00000E+00, 2.00000E+00, 4.00000E+00, 2.00000E+00, 6.00000E+00, 2.00000E+00, 3.00000E+00, 7.00000E+00), r=c(2.70000E+01, 1.80000E+01, 2.36000E+02, 1.29000E+02, 9.80000E+01, 5.40000E+01, 1.87000E+02, 2.75000E+02, 6.80000E+01, 1.08000E+02, 1.90000E+01, 6.00000E+00, 5.00000E-01, 3.65000E+01, 1.34000E+02, 1.20000E+01, 3.60000E+01, 1.60000E+01), n=c(4.50000E+01, 4.40000E+01, 3.54000E+02, 3.55000E+02, 1.47000E+02, 1.43000E+02, 4.63000E+02, 4.58000E+02, 2.71000E+02, 2.72000E+02, 3.30000E+01, 3.20000E+01, 1.67000E+02, 1.71000E+02, 1.76000E+02, 9.00000E+01, 6.00000E+01, 5.50000E+01), nObs=1.80000E+01, nStudies=1.00000E+01, nTx=8.00000E+00, baseNObs=2.00000E+00, baseR=c(2.36000E+02, 1.90000E+01, NA), baseN=c(3.54000E+02, 3.30000E+01, NA))

# Covariate

# list(bvl=c(9.57, 9.98, 9.62,9.69,9.3,9.3,9.5,9.5,9.9,10.1,10.26,9.88,8.12,8.25,8.64,8.88,2.04,1.78))

*# study-level covariate values used by David Scott (with study-level covariate values*

*# obtained by computing the weighted average of the arm-level covariate values and # the weights taken to be the arm-level sample sizes)* **list(bvl=c(9.77, 9.77, 9.66,9.66,9.5,9.5,9.5,9.5,10.0,10.0,10.07,10.07,8.19,8.19,8.72,8.72,1.92,1.92))**

#initial 1

list(beta=c( NA, 3.34368E-01, 1.13602E-01, -5.55077E-01, -7.34804E-01, 3.70524E-01, 2.93559E-01, -9.03393E-02), alpha=c(-2.95439E-01, 6.57458E-01, 4.73809E-01, -4.71472E-02, -3.10827E-01, -2.03360E-01, 6.43383E-01, -1.53570E-01, 7.18140E-01, -6.00384E-01), bCov=0)

#initial 2

list(beta=c( NA, 1.12764E-01, 1.32503E-01, 1.66455E-01, -1.15460E-01, -4.17328E-01, 2.10563E-02, -1.87751E-01), alpha=c(1.36943E+00, 1.79207E-01, -7.52895E-01, -5.51611E-01, -1.07020E-01, -1.01341E+00, 3.06775E-01, 8.22929E-02, -2.20997E-01, -5.68833E-02), bCov=1.13602E-01)

# Treatment Coding

# 1 = ETV0.5

# 2= ADV10

# 3 = LMV100

# 4 = Placebo

# 5 = TBV600

# 6 = TDF300

# 7 = IFNA

# 8 = PegIFNA

}
